# Stomatitis, Agranulocytosis, and Multiple Sclerosis in Pregnancy

**DOI:** 10.7759/cureus.95591

**Published:** 2025-10-28

**Authors:** Keyla Payano, Allison Samuel, Kelsey B Bryant

**Affiliations:** 1 Division of General Internal Medicine, Mount Sinai Hospital, New York, USA

**Keywords:** agranulocytosis, drug rection, multiple sclerosis, pregnancy, primary care

## Abstract

While agranulocytosis with stomatitis is not commonly seen in the primary care setting, drug reactions, especially in patients with multiple sclerosis or conditions that require immunomodulating drugs, should be thought of as a possible etiology. We report a case of a 32-year-old pregnant woman with a history of multiple sclerosis presenting with stomatitis, found to have agranulocytosis, in the outpatient setting. The patient had been initiated on ocrelizumab months prior and had an active influenza infection, both of which can be rarely associated with stomatitis and agranulocytosis.

## Introduction

Multiple sclerosis (MS) is a chronic autoimmune disorder with varying presentation and course that affects the central nervous system. Autoimmune destruction of myelin causes symptoms such as muscle weakness, imbalance, loss of vision, and bladder/bowel dysfunction [1]. Agranulocytosis, defined as an absolute neutrophil count (ANC) below 100 neutrophils in 1 microliter of blood, is rare, occurring in 6-8 cases per million people per year [2]. Many medications and infections are associated with agranulocytosis, including chemotherapies, anticonvulsants, bacterial, and viral infections. 

Agranulocytosis frequently presents with infectious symptoms like fevers and chills. It also commonly presents with stomatitis, painful inflammation of the oral mucous membranes [2]. There are numerous etiologies of stomatitis with agranulocytosis, including viral infections such as influenza and drug reactions, notably including monoclonal antibodies for conditions like MS.

## Case presentation

A 32-year-old woman at 11 weeks of gestation (G2P1), with a history of MS on ocrelizumab, and papillary thyroid cancer status post thyroidectomy, presented with five days of a small tongue ulcer and body aches. Aside from the painful lesion on her tongue and body aches, the patient had no specific symptoms. She denied fevers, chest pain, urinary symptoms, diarrhea, or abdominal pain. She reported gum swelling and was evaluated by a dentist the day prior, who recommended follow-up with her primary care physician or a visit to the emergency department for further medical evaluation. Notably, her close contacts had recently recovered from influenza. For treatment of her MS, the patient began ocrelizumab therapy two years before presentation and continued to receive the infusion as indicated every six months. Her most recent infusion was four months prior to presentation, before becoming pregnant.

**Figure 1:**
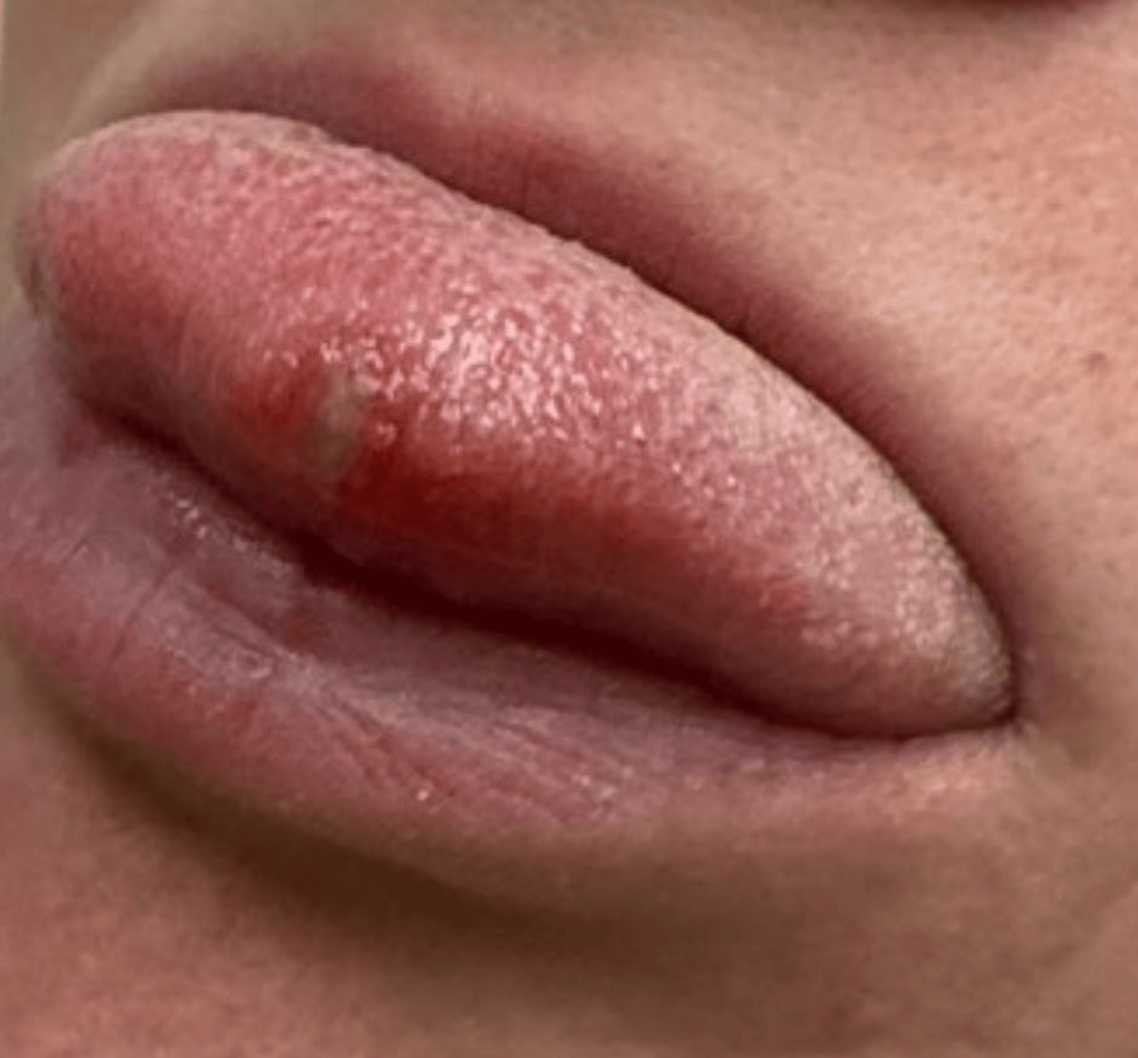
Stomatitis of the Tongue

Physical examination was notable for nasal congestion, cervical lymphadenopathy, and an approximately 0.5 cm erythematous ulcer on the tongue, consistent with stomatitis (Figure 1). Otherwise, the exam was unremarkable. The patient was prescribed oral lidocaine solution, and labs were sent.

The complete blood count returned with an ANC of 0 (Table 1). She was called back for an urgent appointment the following day. Her oral ulcer was slightly larger and was now interfering with speaking, eating, and drinking. She was ultimately referred to the emergency department for emergent evaluation. Although she was clinically stable and looked well on exam, her early pregnancy status and the need for prompt, multidisciplinary input from obstetrics, neurology, and hematology significantly influenced the decision to refer for emergency evaluation. Upon repeat in the emergency department, her hemoglobin and platelets were within normal limits, and her ANC was confirmed to be 0.

**Table 1:** Initial and Confirmatory Complete Blood Count Results

Component	Reference Range	Initial	Follow-Up (24 Hours Later)
White blood cells (10^3^/µL)	3.4-10.8	4.3	4.2
Hemoglobin (g/dL)	11.1-15.9	13.2	13.8
Hematocrit (%)	34.0-46.6	38.0	40.4
Platelets (10^3^/µL)	150-450	287	317
Absolute neutrophils (10^3^/µL)	1.9-8.0	0.0	0.0
Neutrophil (%)	40.0-78.0	0.0	0.9
Lymphocyte (%)	15.0-50.0	58.2	62.7
Monocyte (%)	2.0-11.0	29.6	25.5
Eosinophil (%)	0.0-5.0	0.0	0.0
Basophil (%)	0.0-1.0	2.6	1.8

In the hospital, Hematology was consulted, and a peripheral blood smear was obtained that demonstrated activated monocytes, lymphocytes, acanthocytes, and, notably, no blasts. She was found to be influenza A positive and given oseltamivir. Additional workup, including Epstein-Barr virus, human immunodeficiency virus, cytomegalovirus, toxoplasmosis, antinuclear antibody, and hepatitis A, B, and C, was negative. She was monitored for 48 hours, during which Maternal-Fetal Medicine and Neurology were consulted. Bone marrow biopsy and treatment with granulocyte colony-stimulating factor (G-CSF) were considered; however, given her clinical stability and Neurology’s concern for potential MS exacerbation, G-CSF was deferred. Notably, G-CSF was not thought to be contraindicated in pregnancy. She had several pelvic ultrasounds, all of which were consistent with normal fetal activity and development for gestational age. After interdisciplinary consideration, stomatitis and agranulocytosis were attributed to a late-onset drug reaction to ocrelizumab, with possible contribution from influenza A. She was discharged home with follow-up and given neutropenic precautions. Her ANC slowly increased over several weeks and ultimately normalized (Table 2).

**Table 2:** Discharge and Outpatient Follow-Up Complete Blood Count Results

Component	Reference Range	Day of Discharge (4 Days Later)	Outpatient Follow-Up (6 Weeks Later)
White blood cells (10^3^/µL)	3.4-10.8	4.9	7.1
Hemoglobin (g/dL)	11.1-15.9	13.1	12.2
Hematocrit (%)	34.0-46.6	38.3	37.2
Platelets (10^3^/µL)	150-450	305	260
Absolute neutrophils (10^3^/µL)	1.9-8.0	0.4	4.5
Neutrophil (%)	40.0-78.0	8.9	62.0
Lymphocyte (%)	15.0-50.0	58.1	30.0
Monocyte (%)	2.0-11.0	11.6	6.0
Eosinophil (%)	0.0-5.0	0.9	1.0
Basophil (%)	0.0-1.0	0.9	1.0

## Discussion

Given that agranulocytosis is uncommon, occurring in 6-8 cases per million people per year, it is not frequently seen in the primary care setting [1]. Drug reactions, particularly in patients with MS or on medications that modulate the immune system, should be considered as a potential cause. Ocrelizumab is an anti-CD20 monoclonal antibody targeting CD20-expressing B cells. Ocrelizumab has been associated with hypogammaglobulinemia and neutropenia, ultimately predisposing to severe infections, though this is rare [3]. The FDA Adverse Event Reporting System reports only 25 cases of neutropenia reported among 3,177 patients (<1%) on ocrelizumab [4]. Several case reports have been written demonstrating late-onset neutropenia in patients who received ocrelizumab, many ultimately requiring hospitalization [5]. 

Neutropenia seen with immune-modulating therapies for MS can be acute or late onset. Neutrophils have a rapid turnover, and their depletion may be influenced by drug-associated infections, therapy-related mechanisms, and immune dysfunction [5]. However, the exact mechanism of neutropenia is still widely debated. Incidence of agranulocytosis across drug classes is described in Table 3.

**Table 3:** Causes and Incidence of Agranulocytosis and Stomatitis References [6,7].

Drug Class	Examples	Reported Incidence of Agranulocytosis (%)
Antithyroid drugs	Carbimazole, methimazole, propylthiouracil	16.70
Antipsychotics	Clozapine	0.80
Antibiotics	Beta-lactams, trimethoprim-sulfamethoxazole, cephalosporins	49.30
Antiepileptics	Carbamazepine, phenytoin	11.80
Antiplatelet agents	Ticlopidine, acetylsalicylic acid (aspirin)	6.90
Anti-inflammatory drugs	Sulfasalazine, metamizole (dipyrone)	0.20

Our patient also had influenza A, which is another potential contributor to her agranulocytosis, supported by the timeline of spontaneous resolution. A study by Colamussi et al. points to influenza A infection accelerating neutrophil apoptosis [8]. Our patient was also pregnant, and while agranulocytosis is rare in pregnancy, especially gestational agranulocytosis, it makes the patient and fetus more susceptible to serious illness and even miscarriage [9]. These numerous risk factors contributed to the complexity of establishing the etiology of the patient's presentation in this case, ultimately making the true cause uncertain. Although ocrelizumab was initially suspected, the patient’s spontaneous hematologic recovery without G-CSF and normalization of neutrophils within six weeks is also consistent with influenza-related agranulocytosis. This case highlights the diagnostic challenges when multiple risk factors coexist and underscores the importance of comprehensive evaluation to better attribute causality. 

There are many etiologies of stomatitis, including bacterial or viral infection as well as mechanical or chemical trauma (Table 4). Oral ulcers affect up to 20-25% of the population [10]. ANC of zero in our patient and positive influenza A infection likely contributed to her presentation of stomatitis. Consideration of our patient’s history of MS, prior treatment with ocrelizumab, and acute infection with influenza A was essential in establishing the pathogenesis of her presenting symptoms.

**Table 4:** Causes and Incidence of Stomatitis References [11-13].

Cause	Incidence/Association
Recurrent aphthous stomatitis (RAS)	Affects ~25% of the general population
Nutritional deficiencies	20.9% iron deficiency, 20.1% low hemoglobin, 4.8% Vitamin B12 deficiency, 2.6% folic acid
Stress	Significantly associated with onset of recurrent aphthous stomatitis (OR = 2.72)
Smoking	Protective factor; lower incidence in smokers (AOR = 0.5)
Systemic diseases	Higher risk in patients with diabetes (aOR = 1.6) and rheumatic disease (aOR = 2.1)
Oral trauma	Statistically significant association with recurrent aphthous stomatitis
Use of SLS-containing toothpaste	Statistically significant association with recurrent aphthous stomatitis
Wearing braces/dentures	Statistically significant association with recurrent aphthous stomatitis
Genetic predisposition	Family history significantly associated with recurrent aphthous stomatitis

## Conclusions

Although agranulocytosis and stomatitis are rare complications of ocrelizumab therapy, they should be considered in patients receiving this treatment for MS. In this case, concurrent influenza A infection and pregnancy likely contributed to the development of agranulocytosis and increased her risk for adverse outcomes. Clinicians should remain vigilant for these complications, particularly in pregnant patients, to allow for early recognition and timely intervention. Ultimately, our patient remained clinically stable, did not require G-CSF, and improved with conservative management, while some equipoise remains regarding the true etiology.
